# Deep neural networks with promising diagnostic accuracy for the classification of atypical femoral fractures

**DOI:** 10.1080/17453674.2021.1891512

**Published:** 2021-02-25

**Authors:** Georg Zdolsek, Yupei Chen, Hans-Peter Bögl, Chunliang Wang, Mischa Woisetschläger, Jörg Schilcher

**Affiliations:** a Department of Orthopedics and Department of Biomedical and Clinical Sciences, Faculty of Health Science, Linköping University, Linköping;;; b Department of Biomedical Engineering and Health Systems, Royal Institute of Technology , Stockholm ;; c Department of Orthopedic Surgery, Gävle Hospital ;; d Department of Radiology and Department of Medical and Health Sciences , Linköping ;; e Center for Medical Image Science and Visualization, Linköping University , Linköping ;; f Wallenberg Centre for Molecular Medicine, Linköping University , Linköping , Sweden

## Abstract

Background and purpose — A correct diagnosis is essential for the appropriate treatment of patients with atypical femoral fractures (AFFs). The diagnostic accuracy of radiographs with standard radiology reports is very poor. We derived a diagnostic algorithm that uses deep neural networks to enable clinicians to discriminate AFFs from normal femur fractures (NFFs) on conventional radiographs.

Patients and methods — We entered 433 radiographs from 149 patients with complete AFF and 549 radiographs from 224 patients with NFF into a convolutional neural network (CNN) that acts as a core classifier in an automated pathway and a manual intervention pathway (manual improvement of image orientation). We tested several deep neural network structures (i.e., VGG19, InceptionV3, and ResNet) to identify the network with the highest diagnostic accuracy for distinguishing AFF from NFF. We applied a transfer learning technique and used 5-fold cross-validation and class activation mapping to evaluate the diagnostic accuracy.

Results — In the automated pathway, ResNet50 had the highest diagnostic accuracy, with a mean of 91% (SD 1.3), as compared with 83% (SD 1.6) for VGG19, and 89% (SD 2.5) for InceptionV3. The corresponding accuracy levels for the intervention pathway were 94% (SD 2.0), 92% (2.7), and 93% (3.7), respectively. With regards to sensitivity and specificity, ResNet outperformed the other networks with a mean AUC (area under the curve) value of 0.94 (SD 0.01) and surpassed the accuracy of clinical diagnostics.

Interpretation — Artificial intelligence systems show excellent diagnostic accuracies for the rare fracture type of AFF in an experimental setting.

Atypical fractures occur at atypical locations in the femoral bone and show a strong association with bisphosphonate treatment (Odvina et al. 2005, 2010, Shane 2010, Shane et al. 2010, Schilcher et al. 2011, 2015, Starr et al. 2018). In contrast to the metaphyseal area, which is the site for the majority of all fragility fractures, the diaphyseal region is where atypical fractures occur. As is the case for any other insufficiency-type fracture of the diaphysis, atypical fractures show specific radiographic features, such as a transverse or short oblique fracture line in the lateral femoral cortex and focal cortical thickening (Schilcher et al. 2013, Shane et al. 2014). These features differ from those of normal femur fractures (NFFs), which show oblique fracture lines and no signs of focal cortical thickening (Shin et al. 2016b).

Early and correct diagnosis of AFF is essential for appropriate management (Bogl et al. 2020a), which minimizes the risk of healing complications (Bogl et al. 2020b). In clinical routine practice, conventional radiographs are used to diagnose complete AFF. However, routine diagnostic accuracy is poor, and < 7% of AFF cases are correctly identified in this way (Harborne et al. 2016).

Artificial intelligence (AI), deep learning through convolutional networks, has proven effective in the classification (Russakovsky et al. 2015) and segmentation (Ronneberger et al. 2015) of medical images in general, and for bone fractures in particular (Brett et al. 2009, Olczak et al. 2017, Chung et al. 2018, Kim and MacKinnon 2018, Lindsey et al. 2018, Adams et al. 2019, Urakawa et al. 2019, Kalmet et al. 2020). Given the very specific radiographic pattern of these fractures, AI appears to be a useful tool for finding the needle (AFF) in the haystack (NFF).

We evaluated the abilities of different deep neural networks to discriminate complete AFF from NFF on diagnostic plain radiographs in an experimental setting and we assessed the effect of limited user intervention on diagnostic accuracy.

## Patients and methods

### Dataset and preprocessing procedures

**Figure 1.**
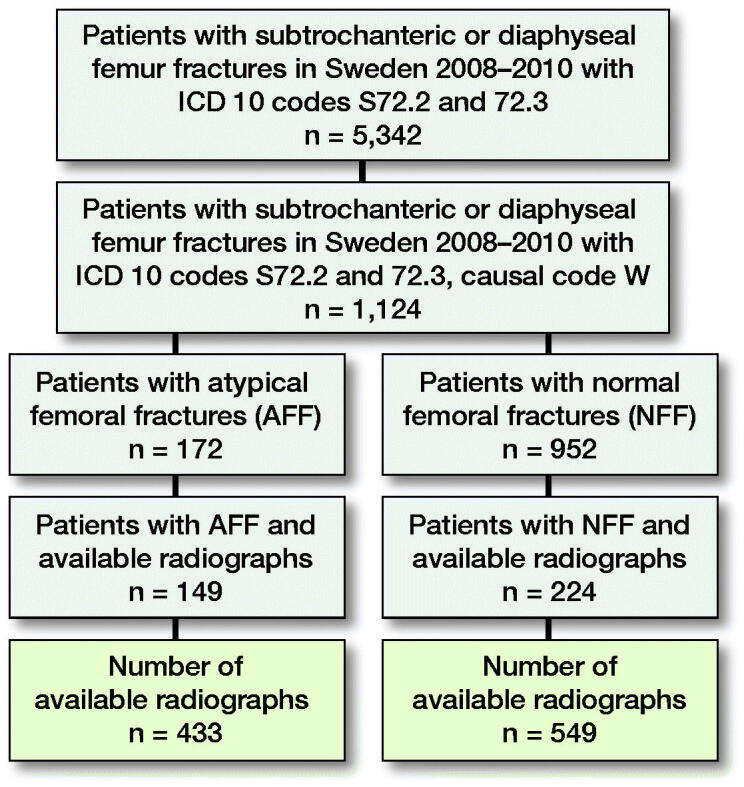
The study cohort recruitment process.

The original dataset of radiographs comprised patients with complete AFFs and NFFs derived from a cohort of 5,342 Swedish women and men aged ≥ 55 years who had suffered a fracture of the femoral shaft in the period 2008–2010 (Figure 1). We extracted and anonymized the radiographs of 151 subjects with AFF and 230 subjects with NFF owing to their accessibility from the research PACS at Linköping University Hospital and excluding all patients with signs of previous surgery, such as joint prosthesis or other orthopedic implants, and signs of pathological fractures (Schilcher et al. 2015). Fracture classification into AFF and NFF was based on repeated individual review in several previous studies with excellent interrater reliability (Schilcher et al. 2011, 2015, Bogl et al. 2020b). For each subject, several radiographs were available. During manual screening, images with extensive artefacts (e.g., splints or plasters) were excluded. The final dataset included 433 radiographs from 149 patients with AFF and 549 from 224 patients with NFF. For the purpose of image processing, the original images were converted from the Digital Imaging and Communication in Medicine (DICOM) format to the Joint Photographic Experts Group (JPEG lossless) format. To allow for transfer learning from ImageNet, grayscale images were converted to Red Green Blue (RGB) images with 3 channels (with identical duplication for each channel). Moreover, all the images were padded to a square size and downsampled to 256 × 256 pixels to reduce the amount of data and computing time for the whole image. Image intensity was normalized to have a mean of 0 and standard deviation of 1. The images were augmented through random rotation (± 10°), shifting (< 10%), and zooming (< 10%) so as to increase the robustness of the trained model. The dataset used in this study will be shared in the “AIDA Dataset Register” (https://datasets.aida.medtech4health.se).

### Network architecture

We identified several network structures that had passed benchmark thresholds for large-scale visual recognition through the ImageNet Large Scale Visual Recognition Challenge (ILSVRC) (Russakovsky et al. 2015). For image classification, convolutional networks are the most widely applied deep neural networks. Convolutional networks build upon convolutional layers, involving the application of a set of machine-learned convolution filters to the input image so as to extract image features such as lines, edges, and corners. During image processing, the filter slides across the image and stacks along the channel’s dimension in the convolutional layer (Goodfellow et al. 2016).

#### VGG

VGG (Visual Geometry Group) is a classic convolutional neural network (CNN) that consists of stacked convolutional layers, pooling layers, and fully connected layers (Simonyan aand Zisserman 2014), connected sequentially. These layers are the building blocks found commonly in modern CNNs. In the ILSVRC 2014 challenge, VGG was ranked in 2nd place (after the Inception network) for the image classification task and in 1st place for the localization task. We used the 19-layer model due to its high performance.

#### Inception network

The Inception network (the first version was also referred to as GoogLeNet) won the ILSVRC 2014 contest with a top-5 error rate of 6.7% (Russakovsky et al. 2015). This is a level close to human perception. Inception consists of 22 convolutional layers with batch normalization. Due to the introduction of inception blocks, each consists of parallel connections of four convolutional pathways. The Inception network uses 20-times fewer parameters than VGG. The computational cost associated with the use of Inception is therefore much lower than that linked to VGG or AlexNet, making it accessible to mobile computing devices with limited computational resources. The Inception architecture has been refined in various ways. We used InceptionV3 (Szegedy et al. 2016), which is 42 layers deep, and the computational cost is only about 2.5-fold higher than that of GoogLeNet (Inception V1), while at the same time being more efficient than VGG.

#### ResNet

The residual neural network ResNet won the ILSVRC 2015 contest (He et al. 2016). The ResNet strategy is based on an attempt to solve the problem of degradation with increasing depth. The network applies a residual function in a residual network based on the hypothesis that optimizing a residual mapping function is easier to achieve than optimizing the original unreferenced mapping. The network converges faster and gains accuracy with increasing depth (He et al. 2016). In a hyper-parameter searching experiment, we applied ResNet with depths of 18, 36, 50, 101, and 152 layers; ResNet50 showed the best performance for the number of images available in our study. Therefore, only the results obtained from ResNet50 are reported here.

### Transfer learning

Transfer learning is a technique that applies known image data to pre-train a deep neural network by reusing image features from other tasks to learn the classification task at hand and, thereby, increase the performance of the neural network. It allows training of the network through random initialization in a situation of limited data and resources. ImageNet, which is currently the largest publicly available dataset for object recognition, is widely applied in transfer learning (Deng et al. 2009). ImageNet is an open image database that consists of 10,000,000 images collected from the internet. These images depict more than 10,000 object categories and every image is labeled according to the category it belongs to. For pre-training, we use the subset from the ILSVRC 2012 image classification challenge. This subset of images contains 1,000 object categories and 1,200,000 images. Even though these images are different from the radiographic images in our study, it is generally believed that pre-training of the CNN networks on this complicated image classification task will allow the networks to learn a hierarchy of generalizable features. Despite the differences between natural images and radiographs, transfer learning from ImageNet can make medical image recognition tasks more effective (Shin et al. 2016a). The cross-modality imaging transferability makes transfer learning in CNN representation from ImageNet popular in various modalities for imaging recognition.

### Feeding pathway

2 diagnostic pipelines were constructed using the CNNs as the core classifier, in order to study the influence of user intervention on network performance.

#### Automated

The radiographs were directly fed into the network with size and intensity normalization steps, as described above. All 3 CNN architectures (VGG19, InceptionV3, and ResNet50) were tested for diagnostic accuracy in relation to the classification of the radiographs to either AFF or NFF.

#### Intervention

**Figure 2.**
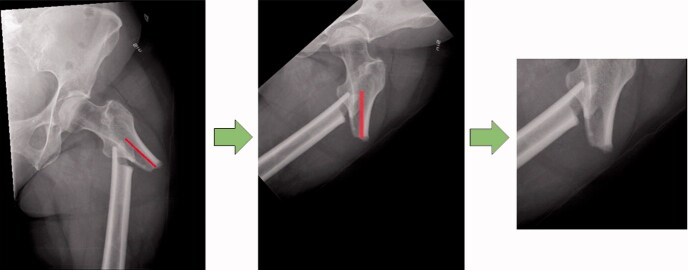
The intervention pathway involves reposition (A), rotation (B), and cropping (C).

The radiographs show the femur in different positions in terms of the rotation and position of the radiograph. An automated script (see Supplementary data) was created using the Keras (https://keras.io/api/preprocessing/image/) and OpenCV package (https://opencv.org/) to move the fracture towards the center of the image and to rotate the femur into the vertical position. In addition, all the images were cropped to a size of 256 × 256 pixels around the center of the fracture (Figure 2). Using this intervention, we attempted to increase the precision of the network in focusing on the features of the fracture. The intervention involves 2 computer mouse clicks per image from the user interface and visual screening to ensure the quality of these images.

### Evaluation methods

We used cross-validation to obtain more accurate results with less bias in the machine learning studies. Theoretically, the dataset is split into K-folds, of which 1 fold is for validation and the other folds are for training. The training and validation processes were repeated several times using different folds each time. The final results were then averaged and the standard deviation (SD) was calculated. We also calculated the diagnostic accuracy, precision, sensitivity, and specificity of each network to discriminate between the 2 types of fractures and present area under the ROC (receiver operating characteristics) curves (AUC) for each network and the 2 feeding pathways. Neural networks typically do not provide insights into the processes within the network that lead to a specific result. We used class activation mapping (CAM) to visualize the features that the network is learning. CAM visualizes a discriminative image region that is used by the network to identify a certain type of fracture (Zhou et al. 2016).

### Experimental setup

During the training process, the batch size was set to 5 and trained for 100 epochs for InceptionV3 and ResNet50. For VGG19, we trained for 200 epochs, since it converged at a slower rate. The learning rate was set to 10^−5^ for all 3 models as a result of fine-tuning. A stochastic gradient descent (SGD) optimizer was used for VGG19. The Root Mean Square Propagation (RMSprop) and Adaptive Moment Optimization (Adam) optimizers were used for InceptionV3 and ResNet50 (Kingma and Ba 2014).

### Ethics and funding

Ethical approval for the study was obtained from the Swedish Ethical Review authority (Dnr. 2020-01108). JS has received institutional support or lecturer’s fees outside of this work from Link Sweden AB, Depuy Synthes, and Sectra.

## Results

### Automated pathway

**Figure 3.**
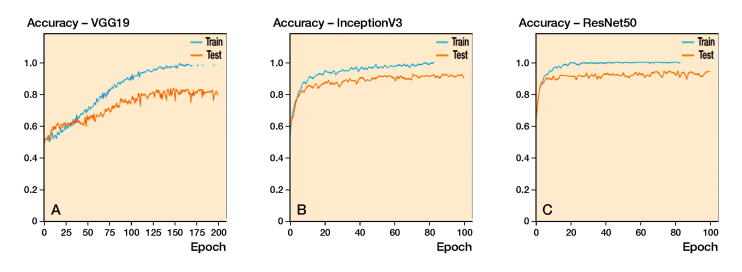
Accuracy plots for: (A) VGG19; (B) InceptionV3; and (C) ResNet50, as expressed for the automated method.

**Figure 4.**
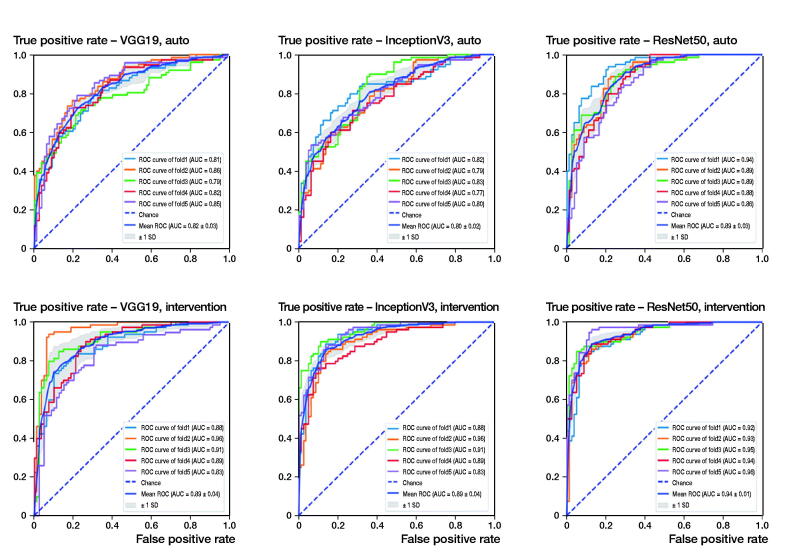
Receiver operating characteristics (ROC) curves for each network and the automated (upper row) and the intervention (lower row) pathway. The intervention pathway of the ResNet50 network shows the lowest rate of false positives at the highest rate of true positives yielding a mean AUC (area under the curve) of 0.94.

When using standardized, pre-processed input data, the evaluated accuracies for the validation dataset were 91% (SD 1.3), 83% (SD 1.6), and 89% (SD 2.5) for ResNet50, VGG19, and InceptionV3. It took about 4 hours to train 1 fold with 100 epochs each on a personal computer with an Intel Core i7 8700 CPU, 16 GB RAM and an NVIDIA GTX 1070 graphic card. According to the accuracy plots (Figure 3) and ROC curves (Figure 4, upper row), ResNet not only achieved the highest final diagnostic accuracy, mean AUC value 0.89 (SD 0.03), but it also reached high levels after fewer iterations compared with the competitor networks. The results of the 5-fold cross-validation are shown in Table 1 and Figure 4 (upper row).

**Table 1.** Cross-validation of the automated method using VGG19, InceptionV3, and ResNet, expressed as percentages and averages with standard deviations (SD) for K-folds

K-fold	VGG19	Accuracy (%) InceptionV3	ResNet
Fold1	81.6	85.3	89.1
Fold2	84.4	89.1	90.7
Fold3	84.4	91.7	90.0
Fold4	81.6	91.1	92.5
Fold5	81.4	90.0	90.0
Average	82.7	89.4	90.5
SD	1.6	2.5	1.3

### Intervention pathway

After adjustment of the image alignment and rotation, the diagnostic accuracies increased to 94% (SD 2), 92% (SD 2.7), and 93% (SD 3.7) for ResNet50, VGG19, and InceptionV3, respectively (Table 2). Similar improvements can be seen in the AUC values of the ROC curves (Figure 4, lower row), showing the highest diagnostic accuracy for the ResNet50 network, mean AUC value 0.9 (SD 0.01).

**Table 2.** Cross-validation for intervention method (manual adjustment of alignment and rotation) using VGG19, InceptionV3, and ResNet expressed as percentages and averages (SD) for K-folds

K-fold	VGG19	Accuracy (%) InceptionV3	ResNet
Fold1	90.4	97.0	94.1
Fold2	94.9	94.3	94.3
Fold3	91.4	91.7	94.2
Fold4	95.1	88.0	97.0
Fold5	89.0	96.2	95.2
Average	92.2	93.4	94.4
SD	2.7	3.7	2.0

### Comparison with multi-metrics

When comparing multiple diagnostic metrics, the ResNet50 network outperformed the competitor networks in terms of specificity (92%, SD 3) and precision (92%; SD 3), whereas the InceptionV3 network showed a slightly higher sensitivity of 91% (SD 3.4), as compared with 89% (SD 3.3) when using the automated pathway. With sensitivity of 94% (SD 1.5), specificity of 96% (SD 1.9), and precision of 96% (SD 1.9), the ResNet50 outperformed all its competitor networks (Table 3).

**Table 3.** Comparison with Multi-Metrics depicting accuracy, sensitivity, specificity, and precision of discrimination between fracture types expressed as percentages (SD) for the automatedmethod and the intervention method for each network

Network	Method	Accuracy	Sensitivity	Specificity	Precision
VGG19	Automated	82.7 (1.6)	85.4 (4.0)	79.6 (4.8)	81.0 (3.4)
Interactive	92.2 (2.7)	93.0 (2.0)	91.6 (5.2)	91.6 (5.2)	
InceptionV3	Automated	89.4 (2.5)	90.6 (3.4)	88.4 (2.6)	88.4 (2.6)
Interactive	93.4 (3.7)	92.8 (5.0)	94.2 (3.7)	94.8 (3.3)	
ResNet50	Automated	90.5 (1.3)	89.0 (3.3)	92.2 (3.0)	92.2 (3.0)
Interactive	94.4 (2.0)	94.4 (1.5)	95.8 (1.9)	95.8 (1.9)	

### Visualization of the results

**Figure 5.**
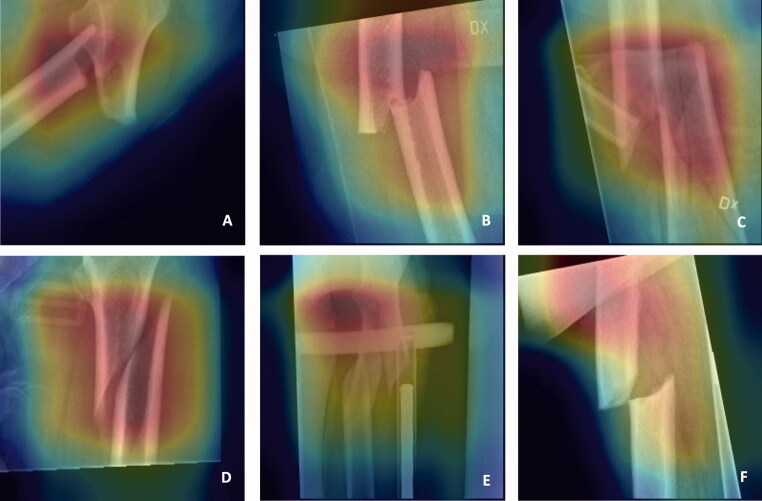
Attention maps showing areas in the image that are utilized by the network for learning through class activation mapping. Fracture region of AFFs (atypical femoral fractures) (A + B) and NFFs (normal femur fractures) (C + D) are correctly depicted by the network. Focus outside the fracture region (E + F) might lead to misclassification.

The final stage was to visualize the features that networks are learning with class activation mapping. Thus, one can observe the discriminative image regions used by the neural networks to identify AFF or NFF. Some examples of the results and potential sources of error in these analyses are shown in Figure 5.

## Discussion

Our aim was to identify deep neural networks that can discriminate atypical femoral fractures from normal femoral shaft fractures on routine conventional radiographs. ResNet50 outperformed other networks with respect to both diagnostic accuracy and time required for the analysis (Table 3). Our findings are in line with those of previous studies indicating that ResNet outperforms other neural networks in classification tasks (He et al. 2016, Brinker et al. 2018). ResNet takes advantage of residual blocks to solve the problem of a vanishing gradient with increasing depth of the neural networks. Moreover, the convergence speed of ResNet is much higher than that of either Inception or VGG19. In line with our hypothesis, the intervention pathway improved the performances of all the networks. In clinical routine, these interventions could be applied by radiographers or other personnel. The smallest increase in benefit was obtained for ResNet, which already showed high accuracy and specificity and precision > 90% in the automated pathway. However, the minimal intervention effort of making 2 computer mouse clicks resulted in an improvement of on average 4%, which in medical diagnostics represents a relevant improvement. Finally, we found class activation mapping to be an essential qualitative tool for allowing human interpretation of the quantitative results given by the network (Figure 5).

**Figure 6.**
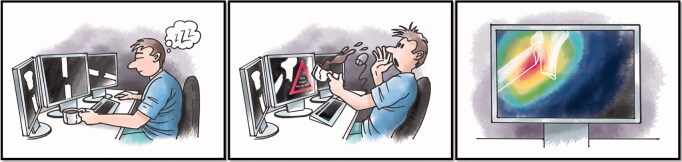
Artificial intelligence designed to attract attention to places where attention is needed. Illustrated by Pontus Andersson.

Studies on AI applications in medical diagnostics typically aim to challenge the power of the human mind in performing highly specialized tasks or tasks of high volume, where processing times matter. In the present study, we used the network to dichotomize, attempting to increase the radiologist’s sensitivity to a specific and rare fracture pattern among a large volume of normal fractures. In our understanding the purpose of the AI is not to replace the clinician but should provide a technical supplement to increase the likelihood of a correct diagnosis. In similarity to approaches taken previously, we used transfer learning from non-medical images to resolve the issue of a limited number of training images and to improve the performance of our CNN (Kim and MacKinnon 2018). Our results are similar to those of a previous study in which a CNN was used to distinguish 695 cases of wrist fracture from 694 non-fracture cases (Kim and MacKinnon 2018), achieving 95% accuracy. Even when CNNs were applied to classify proximal humeral fractures on plain anteroposterior shoulder radiographs, automated distinction of fractured from non-fractured shoulders was achieved with an accuracy of 96%. However, in the same sample, the CNN showed poorer performance in the classification of different types of fractures, with 65%–86% accuracy (Chung et al. 2018). This leads us to conclude that classifying fracture and non-fracture images is an easier task than distinguishing between 2 types of fractures. This type of task is also challenging for the human mind. Previous classification of shoulder fractures into different types according to a well-established classification system (Neer 1970) yielded only approximately 50% inter-observer reliability and 60% intra-observer reliability (Sidor et al. 1993). The human mind tends to suffer from fatigue when engaging in cognitively demanding, repetitive tasks (Tomei et al. 2006, Ren 2018). Therefore, allowing the CNN to perform the bulk analysis and to bring suspected cases to the attention of the radiologist through the visualization of discriminative image regions means that the CNN emerges as an attractive tool in this study (Figure 6).

This study is the first attempt to use artificial intelligence for the radiographic diagnosis of atypical femoral fractures. We used radiographs from a selected cohort of patients who had suffered a femoral shaft fracture but had no previous fractures, pathologic features, or pre-existing implants. This may explain the excellent results and could limit the applicability in a clinical setting, in which the CNN might be blurred by implants and pathologic features in the bone surrounding the fracture site.

In conclusion, we demonstrate that CNNs are a promising tool for the radiographic detection of rare atypical femoral fractures. Given that < 10% of patients are correctly identified on the basis of diagnostic radiographs at the moment, CNNs could contribute potentially as a technical supplement for the clinician, although this is currently limited to the experimental setting. Further training with CNNs and their exposure to a real-world clinical environment are warranted.

## Supplementary Material

Supplemental Material

## Supplementary data

Intervention script is available as supplementary data in the online version of this article, http://dx.doi.org/10.1080/17453674.2021.1891512
