# Supplementary material for: Deep neural networks with promising diagnostic accuracy for the classification of atypical femoral fractures
Source: Acta Orthop. 2021 Feb 25;92(4):394–400. doi: 10.1080/17453674.2021.1891512 (PMC8381921; doi:10.1080/17453674.2021.1891512)
Supplement: Supplemental Material [file IORT_A_1891512_SM2361.pdf]

Supplementary data

Demonstration of preprocessing methods, including cropping, padding, rotation, shifting and zooming.

```
@Author: Yupei Chen
@Date: Jan 13th, 2021

from keras.preprocessing.image import ImageDataGenerator
import cv2

import numpy as np

import os
def generate_data(train_data, train_label, test_data, test_label):
“Generate data for training.

Augmentation through rotation, shifting and zoom.”

if not args.data_augmentation:
print(‘Not using data augmentation.’)

train_datagen = ImageDataGenerator(samplewise_center =
True,
samplewise_std_normalization=True,
validation_split = args.validation_split else:
rescale = 1./255.0) #
print(‘Using real-time data augmentation.’)

train_datagen = ImageDataGenerator(samplewise_center =
True,
samplewise_std_normalization = True,
rescale = 1./255.0, width_shift_range = 0.1, height_shift_
range = 0.1, rotation_range = 10, zoom_range = 0.1) #
validation_split = args.validation_split)

test_datagen = ImageDataGenerator(samplewise_center =
True, s
amplewise_std_normalization = True,
rescale = 1./255.0)
train_datagen.fit(train_data)

test_datagen.fit(test_data)

train_generator = train_datagen.flow(train_data, train_label,
shuffle = True, #

seed = 1,

batch_size = args.batch_size) #
subset = ‘training’)

validation_generator = test_datagen.flow(test_data, test_label,
True
shuffle=False, # False or
seed = 1,
batch_size = args.batch_size) # subset = ‘validation’)
return train_generator, validation_generator
def pad(jpeg_dir, pad_dir):
“crop and padding”
j = 0

for root, dirs, files in os.walk(jpeg_dir):
for name in files:

if name.endswith((‘.jpeg’)):
j += 1

f = os.path.join(root, name) img = cv2.imread(f)

img = np.asarray(img)
shape = np.shape(img)

pad_size = max(shape[0], shape[1])

image = np.zeros((pad_size, pad_size, 3)) #
image[:shape[0],:shape[1]] =
img[:shape[0],:shape[1], :]
        image[:shape[0],:shape[1]] =
img[:shape[0],:shape[1]]
```
